# Options for Synthetic DNA Order Screening, Revisited

**DOI:** 10.1128/mSphere.00319-17

**Published:** 2017-08-23

**Authors:** Diane DiEuliis, Sarah R. Carter, Gigi Kwik Gronvall

**Affiliations:** a Center for the Study of Weapons of Mass Destruction, National Defense University, U.S. Department of Defense, Washington, DC, USA; b Science Policy Consulting LLC, Arlington, Virginia, USA; c Johns Hopkins Center for Health Security, Johns Hopkins Bloomberg School of Public Health, Baltimore, Maryland, USA

**Keywords:** biosecurity, science policy, synthetic biology

## Abstract

Gene synthesis providers affiliated with the International Gene Synthesis Consortium (IGSC) voluntarily screen double-stranded DNA (dsDNA) synthesis orders over 200 bp to check for matches to regulated pathogens and to screen customers. Questions have been raised, however, about the continuing feasibility and effectiveness of screening. There are technical challenges (e.g., oligonucleotides and tracts of DNA less than 200 bp are not screened) and corporate challenges (e.g., the costs of screening are high, but other costs are dropping, so screening is an increasing portion of operating costs). In this article, we describe tangible actions that should be taken to (i) preserve the effectiveness of DNA order screening as a security tool and (ii) develop additional mechanisms to increase the safety and security of DNA synthesis technologies. Screening is not a perfect solution to DNA synthesis security challenges, but we believe it is still a valuable addition to security, and it can remain effective for some time.

## Abstract (precis)

Gene synthesis providers affiliated with the International Gene Synthesis Consortium (IGSC) voluntarily screen double-stranded DNA (dsDNA) synthesis orders over 200 bp to check for matches to regulated pathogens and to screen customers. Questions have been raised, however, about the continuing feasibility and effectiveness of screening.

## COMMENTARY

DNA synthesis is a valuable research tool in the design of new biological products for medicine and manufacturing, and the ability to chemically synthesize long tracts of DNA has allowed for the development of influenza vaccines and diagnostic tests. As with many powerful technologies, however, it is vulnerable to misuse: using DNA synthesis technologies, a nefarious actor would not need direct access to certain pathogens but could chemically synthesize them using sequence information freely available on the Internet. Once synthesized, they could be “booted up” to become infectious. That many viruses can be made from scratch has been demonstrated repeatedly, including in the construction of poliovirus, 1918 influenza virus, and most recently, the virus that causes horsepox (1).

Over the past decade, measures have been taken to reduce the likelihood of misuse. Several commercial suppliers of double-stranded DNA (dsDNA), in the face of uncertain legal liability concerns if their products were linked to biological weapons, joined together in 2009 to create the International Gene Synthesis Consortium (IGSC) (http://www.genesynthesisconsortium.org). IGSC companies work together to develop protocols for individual companies to screen ordered sequences as well as to verify customers. The U.S. Department of Health and Human Services (HHS) also released guidance in 2010 for DNA suppliers, “Screening Framework Guidance for Providers of Synthetic Double-Stranded DNA” (2). HHS recommended that DNA synthesis companies screen ordered sequences of dsDNA in excess of 200 bp and additionally screen the customer to ensure legitimacy. IGSC procedures exceed the HHS guidance; IGSC consists of 11 DNA providers from all over the world, representing 80% of the market, and these companies screen orders against a database that includes U.S. regulated pathogens (select agents) (3), Australia Group list agents (4), U.S. Commerce Control List (CCL) controlled sequences (5), and European Union (EU) sequences. The international organization is still expanding; in the past 2 years, BGI (China) and Bioneer (Korea) became members.

## THE HHS SCREENING GUIDANCE IS BECOMING OBSOLETE

The HHS Guidance (2) is an important, though voluntary, tool in the governance of the fast-paced synthetic biology industry. In addition to raising barriers to nefarious use, it sets a standard of practice that defines responsible behavior among dsDNA providers. It also may be a useful tool for biosafety, if it prevents cases of unintentional or ill-considered ordering of genes from dangerous pathogens, without consideration of potential risks.

Screening dsDNA orders was always a partial solution to potential misuse of DNA synthesis. Commercial suppliers are more likely to rapidly produce high-quality dsDNA for their customers, but they are not the only way to obtain dsDNA; it is possible to synthesize long tracts of dsDNA with commonly available laboratory reagents and equipment. However, in recent years, the HHS Guidance and screening dsDNA orders are increasingly facing serious challenges to their relevance and impact (6). One challenge is its cost to companies: costs for DNA synthesis continue to decrease, while screening remains relatively constant, making screening costs an increasingly larger percentage of total costs. In particular, some orders are not clearly problematic but require a highly trained person to make a judgment about proceeding; these ambiguous orders make up a majority of sequence screening costs (6). Companies that screen risk becoming uncompetitive.

A broader challenge to the HHS guidance is that it does not capture single-stranded DNA (oligonucleotides), which can be used to synthesize genes and even whole genomes (7). Although some laboratory know-how is required to synthesize genes and thus whole pathogens from oligonucleotides, this process is also becoming more straightforward, with tools and products readily available. Oligonucleotides are used ubiquitously in modern research laboratories for many different purposes and are synthesized quickly and cheaply at a much larger scale than dsDNA. The financial and technical challenges for screening oligonucleotides would be immense and cost-prohibitive.

Finally, there are challenges due to the international nature of commercial synthesis. Not all gene synthesis companies are members of the IGSC or screen dsDNA orders. There is also the question of to whom commercial suppliers should report suspicious orders to prevent an actor from shopping for a willing supplier. In the United States, a suspicious order could be reported to the FBI Weapons of Mass Destruction (WMD) Directorate (as recommended by the HHS Guidance), but in many countries, there is no appropriate authority to which companies can report suspicious orders.

## CREATIVE POLICY SOLUTIONS EXIST

The United States and other nations have a vested interest in preserving the success and longevity of screening dsDNA orders as a biosecurity tool. Addressing this problem will require creative policy solutions, as screening is currently a voluntary measure undertaken by private, international companies and is thus not under the direct control of any one country. An array of potential solutions should be implemented to preserve and expand screening as a norm for the field.

Some activities are already under way in the United States, including efforts to develop more refined databases for synthesis companies to use for screening. Much of the screening costs to commercial suppliers arise from the ambiguous nature of screening DNA sequences against entire genomes of pathogens, which include “housekeeping” genes and sequences with unknown function. Improving the database to well-defined sequences of concern may help with this challenge.

Questions remain about the future costs and U.S. Government (USG) responsibilities to curate a rapidly expanding database. One option could be through one of the U.S. National Laboratories, as they have computing power and machine learning capabilities to make a dynamic database. IGSC members may not consent to using a database for screening that is housed by a government and not in their direct control, and the international array of gene synthesis customers may also object to using a service with ties to a particular government. Perhaps the USG could contract this service to a biosecurity enterprise (academic, nonprofit, or industry) that would function solely to support screening for all members of the IGSC. Endorsing this enterprise with a “good housekeeping” status could also assist more nontraditional providers in using screening tools.

Other options include direct financial support to companies for screening. The U.S. Government could award an infrastructure grant to the IGSC to support screening or to fund activities of the IGSC to make it less burdensome to join or participate (8). The U.S. Government may also require that recipients of federal funding purchase DNA only from IGSC suppliers, which was a recommendation originally made by the National Science Advisory Board for Biosecurity (NSABB) but never adopted. There are already laws about the possession of a select group pathogen within the United States; additional regulatory measures the United States could take would necessarily be limited to the United States and so would have limited reach.

Research is also required to learn how to preserve and improve the success of screening DNA orders. For example, it is unknown how many synthesis orders are flagged for further screening, whether customer screening accomplishes much of the same goals as sequence screening, or how many orders are currently referred to authorities. Customer screening is undeniably important: one provider noted “we need to carefully choose our customers as legitimate purveyors of our products—those with a good track record and commitment to our common values.” (participant at an industry workshop in 2017 held under the Chatham House Rule). Studies are also needed on the reach and nature of DNA synthesis companies that do not screen and whether screening is a deterrent to nefarious actors. The USG could fund pilot projects to the IGSC to screen customers ordering oligonucleotides to determine whether such measures would be feasible.

Additionally, the U.S. State Department should encourage international organizations such as the United Nations (UN), World Health Organization (WHO), and Organisation for Economic Co-operation and Development (OECD) to provide support and incentives for screening. This could promote the development of biosecurity tools as well as stimulate international dialogue that may contribute to greater international awareness and consensus building and to further the norm of screening. Finally, on a grand scale, the USG and international partners should investigate supporting an international secretariat, competed and awarded to an entity which could collect dues from member countries. Those member funds could support the database and activities devoted to biosecurity policy concerns. Models for such an arrangement currently exist (the Convention on Biological Diversity [https://www.cbd.int/secretariat/default.shtml] and Scientific Collections International [http://scicoll.org/organization.html]).

## CONCLUSION

The screening of dsDNA orders is not a panacea for biosecurity concerns: it is possible for nefarious actors to work around the screening. However, we believe that screening dsDNA orders still raises barriers to the development of biological weapons and may offer some protection against biosafety concerns. There are a variety of creative solutions that should be explored, which could preserve and improve dsDNA screening as a biosecurity tool, as well as avoid unnecessary burdens on the provider community, which has proven willing and able to cooperate and share responsibility. We urge the policy community to explore these and other potential options, to preserve dsDNA order screening as a biosecurity and biosafety tool for as long as possible, as well as research ways to improve it.
